# Food/Non-Food Classification of Real-Life Egocentric Images in Low- and Middle-Income Countries Based on Image Tagging Features

**DOI:** 10.3389/frai.2021.644712

**Published:** 2021-04-01

**Authors:** Guangzong Chen, Wenyan Jia, Yifan Zhao, Zhi-Hong Mao, Benny Lo, Alex K. Anderson, Gary Frost, Modou L. Jobarteh, Megan A. McCrory, Edward Sazonov, Matilda Steiner-Asiedu, Richard S. Ansong, Thomas Baranowski, Lora Burke, Mingui Sun

**Affiliations:** ^1^Department of Electrical and Computer Engineering, University of Pittsburgh, PA, United States; ^2^Hamlyn Centre, Imperial College London, London, United Kingdom; ^3^Department of Foods and Nutrition, University of Georgia, Athens, GA, United States; ^4^Section for Nutrition Research, Department of Metabolism, Digestion and Reproduction, Imperial College London, London, United Kingdom; ^5^Department of Health Sciences, Boston University, Boston, MA, United States; ^6^Department of Electrical and Computer Engineering, University of Alabama, Tuscaloosa, AL, United States; ^7^Department of Nutrition and Food Science, University of Ghana, Legon-Accra, Ghana; ^8^USDA/ARS Children's Nutrition Research Center, Department of Pediatrics, Baylor College of Medicine, Houston, TX, United States; ^9^School of Nursing, University of Pittsburgh, Pittsburgh, PA, United States; ^10^Department of Neurosurgery, University of Pittsburgh, Pittsburgh, PA, United States

**Keywords:** egocentric image, wearable device, technology-based dietary assessment, low- and middle-income country, artificial intelligence

## Abstract

Malnutrition, including both undernutrition and obesity, is a significant problem in low- and middle-income countries (LMICs). In order to study malnutrition and develop effective intervention strategies, it is crucial to evaluate nutritional status in LMICs at the individual, household, and community levels. In a multinational research project supported by the Bill & Melinda Gates Foundation, we have been using a wearable technology to conduct objective dietary assessment in sub-Saharan Africa. Our assessment includes multiple diet-related activities in urban and rural families, including food sources (e.g., shopping, harvesting, and gathering), preservation/storage, preparation, cooking, and consumption (e.g., portion size and nutrition analysis). Our wearable device (“eButton” worn on the chest) acquires real-life images automatically during wake hours at preset time intervals. The recorded images, in amounts of tens of thousands per day, are post-processed to obtain the information of interest. Although we expect future Artificial Intelligence (AI) technology to extract the information automatically, at present we utilize AI to separate the acquired images into two binary classes: images with (Class 1) and without (Class 0) edible items. As a result, researchers need only to study Class-1 images, reducing their workload significantly. In this paper, we present a composite machine learning method to perform this classification, meeting the specific challenges of high complexity and diversity in the real-world LMIC data. Our method consists of a deep neural network (DNN) and a shallow learning network (SLN) connected by a novel probabilistic network interface layer. After presenting the details of our method, an image dataset acquired from Ghana is utilized to train and evaluate the machine learning system. Our comparative experiment indicates that the new composite method performs better than the conventional deep learning method assessed by integrated measures of sensitivity, specificity, and burden index, as indicated by the Receiver Operating Characteristic (ROC) curve.

## Introduction

More than one-third of low- and middle-income countries (LMICs) face the double burden of malnutrition: undernutrition and obesity, particularly in sub-Saharan Africa, South and East Asia, and Pacific regions (Hawkes et al., 2020; Nugent et al., 2020; Popkin et al., 2020; Wells et al., 2020). The impact of malnutrition includes impaired childhood development, overweight and obesity, and increased risk of chronic diseases. Since malnutrition leads to low productivity, reduced or lost wages, and higher medical expenses, nutrition interventions have been recommended to tackle malnutrition from multiple perspectives in these countries. To conduct nutrition interventions effectively, it is desirable to develop a tool to monitor the nutritional status and to evaluate the impact of interventions at the individual, household, and community levels. However, commonly used dietary assessment methods, such as 24-h recall and food frequency questionnaire, are labor-intensive and highly subjective. These approaches are based on participants' memory and ability to measure food weight/volume and are not suitable for children and adults with low literacy in LMICs (Kristal et al., 1997; Baxter et al., 2008; Sharman et al., 2016). In addition, numerous reports indicate that these methods are biased in different types of food intake (Sharman et al., 2016; Tugault-Lafleur et al., 2017); moreover, completing these assessments can be burdensome for the individual.

Recently, sensor-based dietary monitoring approaches have been used to conduct objective dietary assessment (Hassannejad et al., 2017; Doulah et al., 2019; Imtiaz et al., 2019; Bell et al., 2020). Inertial sensors, proximity sensors, and piezoelectric sensors have been used to monitor body motion, such as arm gestures during eating and chewing/swallowing (Zhang et al., 2009; Li et al., 2013; Thomaz et al., 2017). Microphones have also been used to detect chewing or swallowing sounds (Sazonov et al., 2008; Fontana and Sazonov, 2013; Fukuike et al., 2015; Papapanagiotou et al., 2017). These wearable sensors can measure certain variables related to eating behavior, such as eating episodes and chewing frequencies, but identifying non-eating activities (e.g., talking, smoking) and excluding them from further analysis have been a challenging problem. In addition, only limited food properties (e.g., chewing difficulty) can be assessed from the data recorded by these sensors since it is almost impossible to know the specific food being consumed. In another approach, smart phones or wearable cameras have been used to take pictures of food with or without manual control (Vu et al., 2017; Min et al., 2018; Doulah et al., 2019; Bell et al., 2020). The recorded images contain rich information about food, such as its contents and the time of consumption. These images can also be used to estimate food volume computationally if certain reference information (e.g., a checkerboard card) is present (Thompson and Subar, 2001; Boushey et al., 2009). However, the method using a smartphone relies on the user's memory and motivation to take pictures. In contrast, a wearable camera can record the entire process of food-related behaviors continuously. Information about the eating environment (e.g., home, restaurant, family eating, social gathering etc.) can also be recorded. Besides dietary intake, food sources (e.g., harvesting and gathering), food preservation and storage, and food preparation are also important components to consider in determining the targets of a nutrition intervention. Wearable cameras become very useful in these cases because of their unique functionality in conducting a dietary assessment in multiple perspectives. Despite this attractive feature, there exists a significant problem in processing image data produced by this dietary assessment method. A wearable device acquires an image sequence at a predefined rate (e.g., taking one image every 2 s). As a result, tens of thousands of images must be reviewed for each day of assessment. Although recently developed Artificial Intelligent (AI) technology will eventually scan the data and extract the desired information automatically, at present AI has not yet been mature enough to understand all food-related activities, especially those in LMICs. As an intermediate solution, we use AI to perform the first step in automatically quantifying dietary intake from images: to classify field acquired image data into binary categories: those that contain food (Class 1) and those do not (Class 0). Once classified, researchers will need only to review the food related images in Class 1. This is a major advance since it saves tremendous effort as eating events are usually a small portion of daily events.

**Figure 1:**
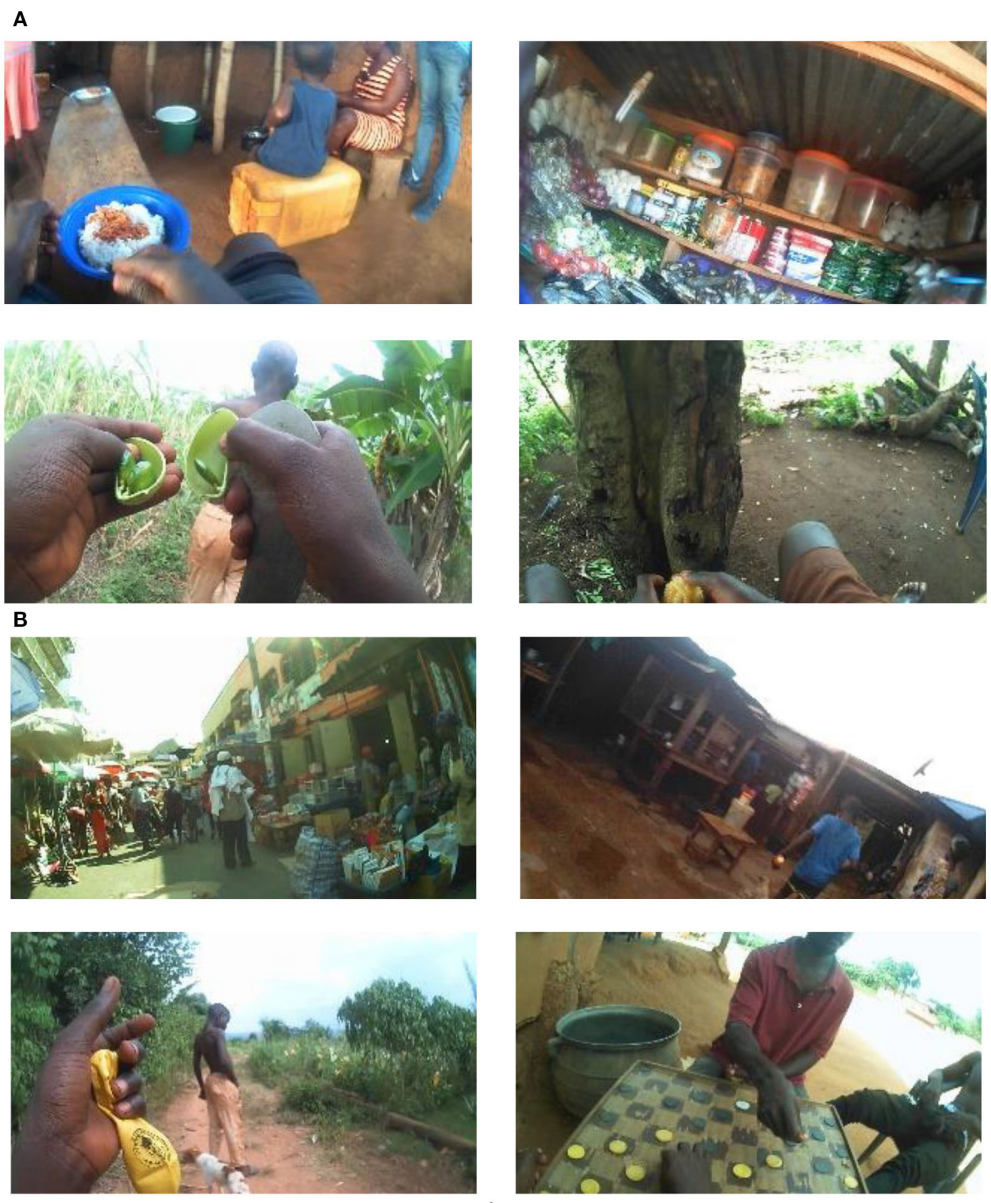
**(A)** Four food-related images, **(B)** four non-food related images.

Food detection from images has been investigated. Traditional image features, including difference of Gaussian (DoG) and bag-of-words, have been used to train a Support Vector Machine (SVM) classifier for food detection (Kitamura and Aizawa, 2009; Farinella et al., 2015). With the advance of AI technology, deep neural networks (DNNs) have been utilized to detect and recognize food from images with improved performance. For example, GoogLeNet and other forms of convolutional neural networks (CNNs) have been applied to food detection (Kagaya et al., 2014; Ragusa et al., 2016). Hossain et al. proposed a novel CNN which can be implemented on mobile devices for real-time application (Hossain et al., 2020). Our team also developed an AI-based method to classify images into food and non-food classes automatically (Jia et al., 2019). However, these existing algorithms are trained using data acquired in the Western world, where people's dietary behaviors and food-related environments (e.g., food sources and preparation procedures) are very different from those in LMICs, as the example images shown in Figure 1 which were acquired in Ghana, Africa. Our evaluation of existing algorithms indicated that they delivered much lower performance due to the higher complexity and diversity in real-world LMIC images.

To meet this challenge, we propose a composite classification architecture with two networks connected by a probabilistic network interface. The first network is a deep neural network which utilizes a large-scale CNN to “understand” each field-acquired image and produce a set of textual tags that describe the image. CNN was chosen among the available deep neural networks because of its exceptional performance in learning complex features in images (Krizhevsky et al., 2012; Farabet et al., 2013; Karpathy and Li, 2015; LeCun et al., 2015; Johnson et al., 2016). The second network is a shallow learning network connected to the first network through a probabilistic network interface, which forms a feature vector by calculating conditional probabilistic measures of food presence from the tags. The shallow learning network then adopts an SVM to classify feature vectors into either food-related or non-food-related class.

The rest of the paper is organized as follows. We describe our food image classification method in section Methods. Our experimental results are presented in section Experimental Results. In sections Discussion and Conclusion, we discuss and conclude our work.

## Methods

**Figure 2:**
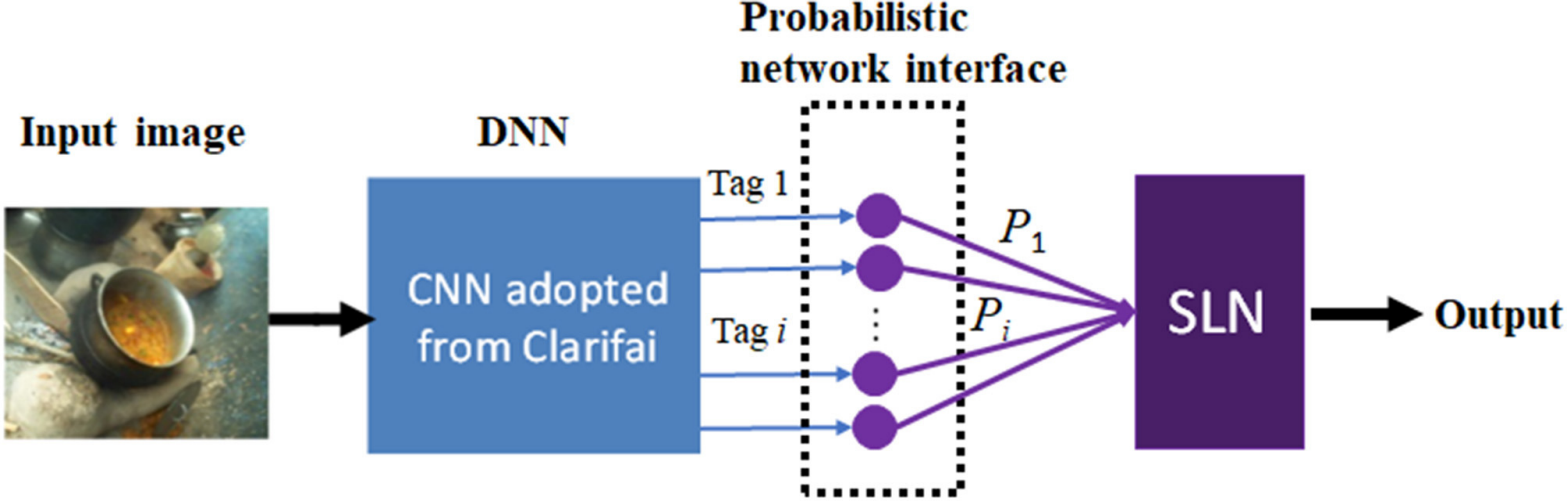
Proposed composite machine learning architecture, which includes a deep neural network (DNN) developed by Clarifai, a probabilistic network interface, and a shallow learning network (SLN).

We propose a composite machine learning architecture that includes a deep neural network (DNN), a probabilistic network interface layer, and a shallow learning network (SLN), as shown in Figure 2. The DNN and SLN are trained separately using different datasets. Specifically, an advanced DNN developed by Clarifai (New York, NY) was adopted. This DNN consists of a large-scale convolutional neural network for annotating images (Zeiler and Fergus, 2014; Clarifai Inc., 2020). The network is trained using an extremely large set of generic images available in the public domain. Therefore, utilizing this well-trained general-purpose DNN solved our problem of labeling and training using a large image set. For each input image, the Clarifai DNN outputs a list of annotation tags (up to 11,000 items) with associated likelihood values. These textual tags, which are mostly nouns but occasionally adjectives in the English language, provide explanations of the input mage. In our approach, the complex machine learning from generic image contents is converted to a set of linguistic descriptors which explain our field-acquired images.

The probabilistic network interface provides a seamless link between the DNN and the SLN. The input of this interface is a list of selected textual tags and their likelihood values produced by the DNN, and its output is a vector of conditional probabilistic measures quantifying the food-prediction power of each tag. This feature vector is then passed on to the SLN, which can be selected from a number of high-performance classifiers, such as the SVM classifier. The operation and mechanism of the probabilistic network interface layer and the SLN structure are explained in detail as follows.

The construction of the probabilistic network interface layer is described as follows. Given an image as the input, the DNN outputs a list of annotation tags w {Tag 1, Tag 2, ⋯ , Tag *M*} with corresponding likelihood values {*v*_1_, *v*_2_, …, *v*_*M*_}, each representing the confidence for a tag. The likelihood value *v*_*i*_ (*i* = 1, …, *M*) is defined as the probability for Tag *i* to be a correct description of the given image: *v*_*i*_ ≡ *P*(*T*_*i*_ = 1), where *T*_*i*_ is a binary random variable which equals 1 when Tag *i* is correctly used to describe the image. The definitions of *v*_*i*_ and *T*_*i*_ also imply *P*(*T*_*i*_ = 0) = 1 − *v*_*i*_. The *v*_*i*_ values are fed to the probabilistic network interface, which calculates the food-prediction power *P*_*i*_ of Tag *i*. Here the food-prediction power *P*_*i*_ indicates the possibility that the food is predicted to be present in the given input image only considering Tag *i*. According to the total probability formula, we have

(1) $$P_{i}=P(Food,T_{i}=1)+P(Food,T_{i}=0)$$

where *P*(*Food, T*_*i*_ = 1) denotes the joint probability of (i) that the given image being predicted (only using Tag *i*) as having “Food” in it and (ii) Tag *i* being a correct description of the given image, and similarly *P*(*Food, T*_*i*_ = 0) denotes the joint probability when *T*_*i*_ = 0. Using the definition of conditional probability, Equation (1) can be further expanded as

(2) $$\begin{array}{l} P_{i}=P(Food\;|\;T_{i}=1)P(T_{i}=1)+P(Food\;|\;T_{i}=0)P(T_{i}=0)\\ \;=P(Food\;|\;T_{i}=1)\;v_{i}+P(Food\;|\;T_{i}=0)(1-v_{i}) \end{array}$$

where *P*(*Food* | *T*_*i*_ = 1) represents the probability for a randomly chosen image to have food in it if the image can be correctly annotated with Tag *i*, and *P*(*Food* | *T*_*i*_ = 0) the probability for the image to have food in it if the image cannot be described by Tag *i*. Since *P*(*Food* | *T*_*i*_ = 1) and *P*(*Food* | *T*_*i*_ = 0) could hardly be calculated directly, we estimate them from a training dataset of the field-acquired images in which each image is labeled as “Food” or “Non-food.” Then *P*(*Food* | *T*_*i*_ = 1) can be approximated by the ratio between (i) the number of images labeled as “Food” and annotated with Tag *i* and (ii) all the images annotated with Tag *i*, while *P*(*Food* | *T*_*i*_ = 0) can be calculated accordingly in a similar manner.

The values of food-prediction powers {*P*_1_, *P*_2_, …, *P*_*M*_} of the selected tags are then fed to the SLN as a feature vector. Based on these features, an SVM with a polynomial kernel is developed to classify the feature vector and thus the input image into one of the two classes: “Food” or “Non-food.” The output of the SVM is the final classification result of the whole learning machine. Note that the results with the SVM will also be compared with those using linear discriminant analysis (LDA) as the SLN. LDA can find the optimal linear combination of features to distinguish two classes, but SVM further supports learning of non-linear models with robust performance.

**Figure 3:**
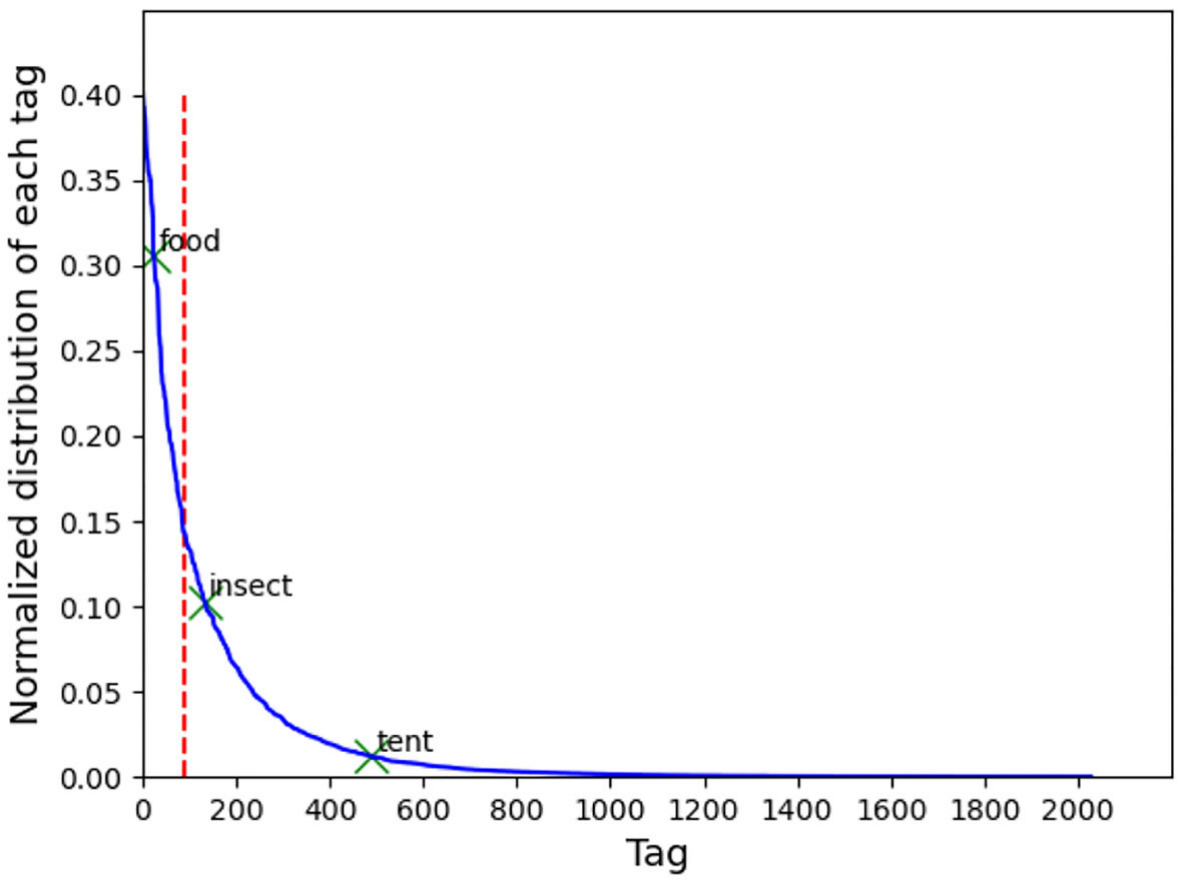
Histogram of the tags representing the occurrence frequency of each tag. The tags on the left side of the vertical line were used to construct the feature vector.

In practice, the number of tags produced by the DNN can be as large as 11,000, resulting in a very high feature dimension for the SLN. Considering the cost and time to acquire and label large image dataset in our field study, the size of training dataset must be limited. Thus, the number of tags, i.e., the dimension of the feature vector, must be decreased to avoid overfitting. In our experiment, the number of tags for each image is limited by Clarifai to 200, but when all the tags in our training set are combined, a total of more than 2,000 tags can be obtained (see Figure 3). This is still a large dimension compared to the size of the training dataset, usually of some thousands of images. So only the most-frequently appearing tags, which account for 50% of the occurrences of all tags, were used in the experiment.

## Experimental Results

### Data Acquisition and Labeling

A multinational research team conducted a field study in Ghana to measure food and nutrient intake of local residents using multiple innovative technologies, including eButton, a wearable device developed in our laboratory (Sun et al., 2015; Jobarteh et al., 2020). This device is equipped with a wide-angle camera worn on the participant's chest using a pin or a lanyard. The camera is tilted downwards in an appropriate angle to take images of food and other food-related objects and activities. In this particular dietary study, the eButton was configured to record approximately 900 images an hour. All the images were saved in the microSD card inside the device and uploaded to a computer or cloud server at the end of each day. Households comprising one or more children qualified for this study. Informed consent was obtained from one of the adults in each household. This experiment used data from nine participants living in a Ghanaian rural community. Each participant wore the eButton for 2 days during waking hours.

The recorded images were reviewed and labeled (“Class 1” or “Class 0,” i.e., “Food” or “Non-food”) manually by researchers as the ground truth. Images containing any food-related information, such as food shopping, gathering and harvesting, food storage and preservation, cooking, and food preparation and consumption were all labeled as food-related images.

### Experimental Procedure and Results

First, an image dataset was constructed containing 59,448 images, in which 8,243 were food images and 51,205 were non-food images. Second, 200 tags and their likelihood values were generated for each image using the DNN (Figure 2). For example, a list of tag and likelihood pairs {(“people”,0.997), (“woman”,0.966), (“adult”,0.956), (“food”, 0.942), …} was generated for the upper left image in Figure 1A. Third, the available images were randomly divided into two datasets, a training set containing 75% of all images and a test set containing the remaining images. Then, in the training set, the number of non-food images was reduced (by randomly removing non-food images) to the same number of food images in order to balance the degree of training for the two classes. Next, the feature vectors of conditional probabilistic measures, i.e., outputs of the probabilistic network interface (Figure 2), were calculated using Equation (2) for all training images to train the SLN (here an SVM classifier). Last, the testing images were used to evaluate the performance of the proposed method.

In addition to the commonly used performance measures, such as True Positive (TP), True Negative (TN), False Positive (FP), False Negative (FN), Sensitivity, and Specificity, we defined a burden index to represent the ratio between the total number of predicted positive images (sum of true positive and false positive) and the total number of images. The burden index, denoted by *B*, is a measure of human workload (i.e., *B* = 0% means “fully automatic” and *B* = 100% means “fully manual”), indicating the percentage of images that need to be reviewed and annotated manually by researchers after the automatic processing.

We also used the DNN alone (without the SLN structure) for food detection and classified each image as food or non-food image by identifying whether the likelihood value of “food”–an explicit tag produced by the CNN model of Clarifai–was larger than a pre-defined threshold. This threshold was a parameter empirically chosen as the borderline value separating food and non-food images. For example, when the threshold was set to 0.7, the input image was classified as a food-related image if the likelihood value of “food” was larger than 0.7. Although a higher sensitivity could have been achieved if a smaller threshold was chosen, the burden index increased rapidly, diminishing the benefits of using automatic classification. As shown in Table 1, our “DNN + SLN” classifier performed better in terms of the overall measures of sensitivity, specificity, and burden index.

**Table 1:** Comparison of classification results using different approaches.

**Method**	**TP**	**FN**	**TN**	**FP**	Sensitivity^*^	Specificity^*^	Burden index B^*^ (%)
DNN + SLN	1,750	311	10,545	2,256	0.85	0.82	27.0%
DNN (Clarifai, threshold = 0.7)	1,296	765	10,400	2,401	0.63	0.81	24.9%
DNN (Clarifai, threshold = 0.6)	1,474	587	9,281	3,520	0.72	0.73	33.6%
DNN (Clarifai, threshold = 0.5)	1,630	431	7,929	4,872	0.79	0.62	43.7%
DNN (Clarifai, threshold = 0.4)	1,756	305	6,491	6,310	0.85	0.51	54.3%
Previous algorithm (*k* = 2)	1,854	207	885	11,916	0.90	0.07	92.7%
Previous algorithm (*k* = 3)	1,469	592	2,751	10,050	0.71	0.21	77.5%
Previous algorithm (*k* = 4)	955	1,106	5,456	7,345	0.46	0.43	55.8%

* Sensitivity = $\frac{TP}{TP+FN}$, Specificity = $\frac{TN}{TN+FP},\;$B = $\frac{TP+FP}{TP+TN+FN+FP}$*100%.

In our previous work (Jia et al., 2019), we studied the classification of eButton images acquired in the United States. In that study, we calculated the total number of food-related tags in each image and defined it as the “evidence value.” If this value was higher than a threshold *k*, the input was classified as a food image. For comparison, we also included the results using our method from Jia et al. (2019) (referred to as “previous algorithm”) in Table 1. It can be seen that the specificity values are much lower than the new method. It demonstrates that our previous algorithm, which delivered acceptable performance for Western-world images, does not work well on the images from LMICs, mainly because of the considerable differences in food sources and preparation/cooking/eating environments.

**Figure 4:**
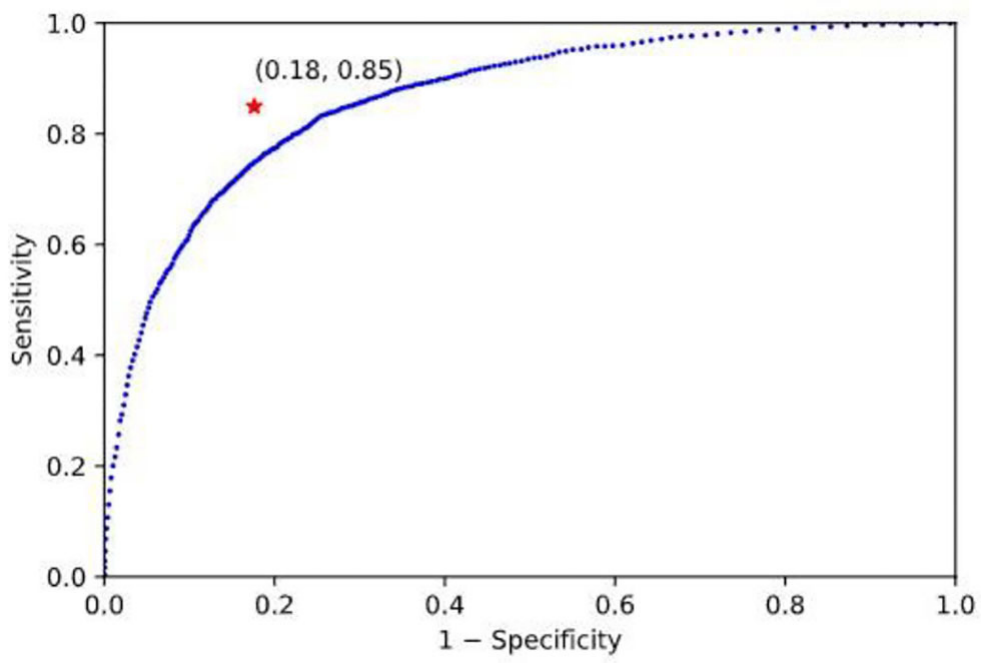
ROC curve of linear discriminative analysis: The blue curve and the red star, respectively, represent the results of the LDA and the SVM classifier. These results indicate that the SVM classifier performs better than the LDA because the red star at (0.18, 0.85) is closer to the ideal point (0, 1).

As another comparison, the linear discriminant analysis (LDA) method was used in the SLN (replacing the SVM) and applied to the same dataset. In order to map the output of classifier to a binary decision (“Food” or “Non-food”), a threshold must be used. By changing the threshold, a Receiver Operating Characteristic (ROC) curve was obtained (Figure 4). Note that an ROC curve plots Sensitivity vs. (1− Specificity) at different classification thresholds. It can be observed that the SVM classifier provides the best performance because the red star at (0.18, 0.85) is closest to the ideal point (0, 1) which represents 100% sensitivity and 100% specificity.

To further verify the robustness of our algorithm constrained by limited availability of data, we repeated the training and testing processes five times randomly using the same set of input data. In each trial, the system was trained (starting from scratch) utilizing randomly assigned images from the dataset, and then tested using the remaining data. After the five trials, the sensitivity and specificity values were averaged with the standard deviation values calculated. The results of the new DNN + SLN method and the DNN only method with four choices of threshold values are shown in Table 2. It can be observed that all standard deviations are reasonably small, indicating the robustness of our experimental results.

**Table 2:** Averaged performances in five random trials (mean ± standard deviation).

**Method**	**Sensitivity**	**Specificity**	**Burden index *B*(%)**
DNN + SLN	0.856 ± 0.014	0.831 ± 0.005	26.3% ± 0.4%
DNN (Clarifai, threshold = 0.7)	0.637 ± 0.007	0.819 ± 0.004	24.4% ± 0.3%
DNN (Clarifai, threshold = 0.6)	0.723 ± 0.008	0.730 ± 0.003	33.2% ± 0.3%
DNN (Clarifai, threshold = 0.5)	0.798 ± 0.007	0.625 ± 0.003	43.3% ± 0.2%
DNN (Clarifai, threshold = 0.4)	0.858 ± 0.006	0.514 ± 0.003	53.8% ± 0.3%

## Discussion

**Figure 5:**
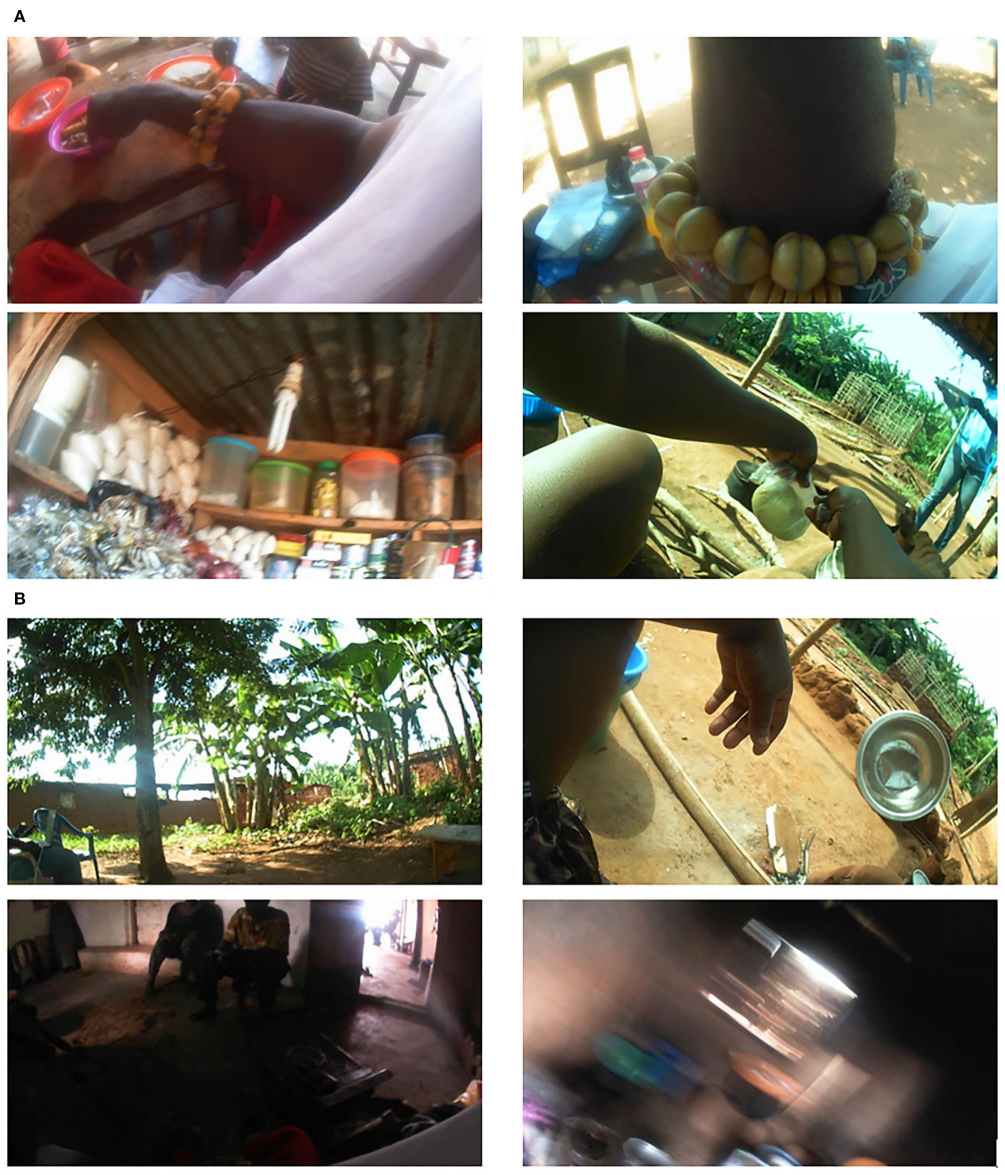
Examples of: **(A)** food images misclassified as non-food images; **(B)** non-food images misclassified as food images.

Detecting food from field-acquired egocentric images in LMICs is a very challenging problem due to complexity and diversity of image contents. Due to the differences in culture and socioeconomical infrastructure between the Western world and LMICs, there are significant differences in food sources and preparation/cooking/eating environments. Therefore, image analysis methods developed for the Western world often fail in processing LMIC images. In many cases, the scenes with and without food are quite similar since food often covers only a very small portion of the whole image, as exemplified by the lower right image in Figures 1A and 5A. In images containing activities of food shopping and harvest/collection (e.g., lower left image in Figures 1A and 5A), small edible items are easy to miss even for humans. Image rotation (see Figure 5B) was common due to variations in body orientation/movement while the participant was performing various activities, such as childcare or serving food for family members. In addition, indoor illumination is often a very significant problem in LMICs, especially in the evenings, which results in dark and blurry images with poor quality. These practical issues increase the difficulty of the food detection problem. Figure 5 illustrates eight images misclassified by our algorithm.

A popular approach to detect or recognize objects from images is to use advanced convolutional neural networks, such as ALexNet (Krizhevsky et al., 2012), GoogLeNet (Szegedy et al., 2015), and ResNet (He et al., 2016). These networks have extraordinary abilities to classify images. Almost concurrently as these networks were developed, another class of networks, such as R-CNN (Girshick, 2015), Faster R-CNN (Ren et al., 2015), and YOLO (Redmon et al., 2015) emerged, capable of not only classifying but also detecting objects by finding a bounding box around each object. Although these two classes of networks are excellent tools to solve our food detection problem, they all require training by a large number of images, and the number increases proportionally to the number of objects. This imposes a high constraint because human food includes tens of thousands (if not more) forms, and the images required for network training could be astronomical. In this paper, we solve the food detection problem by developing a composite machine learning approach built upon two advanced concepts. First, we use the concept of semantic integration (Noy, 2004; Mountantonakis and Tzitzikas, 2019). A pre-trained CNN (Clarifai) produces individual textual tags. The set of tags forms linguistic descriptors of the input image as a whole, instead of individual objects. As a result, diet related activities are described by not only individual edible items, but also a number of related non-edible items such as tables, stools, cookware, utensils and even the gender of humans. Second, we utilize the concept of transfer learning, which solves one problem and applies the knowledge to a different but related problem (Yosinski et al., 2014). We implement the transfer learning concept by developing a novel probabilistic network interface followed by a shallow learning network (SLN). The input to the interface is the output of a DNN which was already trained using images in the Western world with significant differences from those in LIMCs. But the knowledge of the DNN is transferred to the LIMC images by the subsequent network structure which uses the set of linguistic descriptors to both confirm and predict edible items in the input image in a statistical sense (Equations 1, 2). The two concepts implemented by our approach greatly improved deep learning performance with limited input data. We believe that our new network structure provides the AI research community with a new tool for not only detecting edible items in images, but also solving a class of practical problems where training data are limited. We also believe that the proposed machine learning approach can be improved further by exploiting more semantic information in the input image, which we are still working on.

## Conclusion

We have proposed a composite machine learning approach to detect food-related images from large amounts of egocentric images acquired from LMICs. Our composite approach consists of two inter-connected learning networks: 1) a well-trained large-scale DNN that produces a set of textual tags of the input image, and 2) a SLN with a probabilistic network interface layer to integrate the information provided by the tags to classify the input image. Our comparative experiments with challenging real-world images acquired from Africa have produced significantly greater improved performance than the conventional approaches.

## Data Availability Statement

The datasets presented in this article are not readily available because raw images contain identifiable information of research participants. Requests to access the datasets should be directed to the corresponding author.

## Ethics Statement

The protocol of the study involving human participants was reviewed and approved by the Human Subjects Institutional Review Board of the University of Georgia, USA, and the Ethics Committee of Noguchi Memorial Institute for Medical Research of the University of Ghana, Ghana. Written informed consent to participate in this study was provided by the participants or their legal guardian. Written informed consent was obtained from the individual(s), and minor(s)' legal guardian, for the publication of any potentially identifiable images or data included in this article.

## Author Contributions

MS, Z-HM, and WJ were responsible for overall methodological design. GC, YZ, WJ, Z-HM, and MS contributed to the implementation of the image processing algorithms and the evaluation of the experimental results. GC, WJ, Z-HM, TB, BL, MM, and MS contributed to final drafting and editing of the manuscript. BL, GF, ES, AA, MM, MJ, MS-A, RA, TB, WJ, and MS contributed to the design of the field study and the collection of images in Ghana. All authors contributed to the article and approved the submitted version.

## Conflict of Interest

The authors declare that the research was conducted in the absence of any commercial or financial relationships that could be construed as a potential conflict of interest.
